# Development of an *in silico* method for the identification of subcomplexes involved in the biogenesis of multiprotein complexes in *Saccharomyces cerevisiae*

**DOI:** 10.1186/s12918-017-0442-0

**Published:** 2017-07-11

**Authors:** Annie Glatigny, Philippe Gambette, Alexa Bourand-Plantefol, Geneviève Dujardin, Marie-Hélène Mucchielli-Giorgi

**Affiliations:** 1 Institute for Integrative Biology of the Cell (I2BC), CEA, CNRS, Université Paris Sud, Université Paris-Saclay, Avenue de la Terrasse, 91198 Gif-sur-Yvette, France; 2 Université Paris-Est, LIGM (UMR 8049), CNRS, ENPC, ESIEE, UPEM, 77454 Champs-sur-Marne, France; 3 Sorbonne Universités, UPMC Univ Paris 06, UFR927, F-75005 Paris, France

**Keywords:** Protein-protein interactions, PPI network, Graph clustering, Protein complex, Subcomplex, Complex assembly

## Abstract

**Background:**

Large sets of protein-protein interaction data coming either from biological experiments or predictive methods are available and can be combined to construct networks from which information about various cell processes can be extracted. We have developed an *in silico* approach based on these information to model the biogenesis of multiprotein complexes in the yeast *Saccharomyces cerevisiae*.

**Results:**

Firstly, we have built three protein interaction networks by collecting the protein-protein interactions, which involved the subunits of three complexes, from different databases. The protein-protein interactions come from different kinds of biological experiments or are predicted. We have chosen the elongator and the mediator head complexes that are soluble and exhibit an architecture with subcomplexes that could be functional modules, and the mitochondrial *bc*_*1*_ complex, which is an integral membrane complex and for which a late assembly subcomplex has been described. Secondly, by applying a clustering strategy to these networks, we were able to identify subcomplexes involved in the biogenesis of the complexes as well as the proteins interacting with each subcomplex. Thirdly, in order to validate our *in silico* results for the cytochrome *bc1* complex we have analysed the physical interactions existing between three subunits by performing immunoprecipitation experiments in several genetic context.

**Conclusions:**

For the two soluble complexes (the elongator and mediator head), our model shows a strong clustering of subunits that belong to a known subcomplex or module. For the membrane *bc*_*1*_ complex, our approach has suggested new interactions between subunits in the early steps of the assembly pathway that were experimentally confirmed. Scripts can be downloaded from the site: http://bim.igmors.u-psud.fr/isips.

**Electronic supplementary material:**

The online version of this article (doi:10.1186/s12918-017-0442-0) contains supplementary material, which is available to authorized users.

## Background

In silico approaches based on large sets of protein-protein interaction data coming from biological experiments can be used to construct protein-protein interaction networks to model the biogenesis steps of multiprotein complexes in the yeast *Saccharomyces cerevisiae.*

Protein-protein interaction (PPI) networks are built from data coming from several methods but mainly from two-hybrid screens or affinity purification followed by mass spectrometry (for examples see [1–4]). The PPI definition and concepts related to experiments data, as well as the advantages and limitations of the strategies used to identify and characterize PPI are described in [5, 6]. The comparison of large-scale data sets and the different kinds of biases introduced during the experiments is presented respectively in [7, 8]. Proteomic experiments often provide incomplete datasets due to the difficulty for detecting some PPI and to the asymmetry of the some experimental assays. To compensate for these missing data, predicted information that combined structural and non-structural information can be added [9].

All PPI involved in a same biological process can be depicted as a network or graph, where each protein is a node and each interaction an edge between two nodes. Most of these networks are too large to be understandable. Biological information can be then extracted by the means of visualisation tools for the basis of the network representation [10, 11] and by using network analysis tools and clustering algorithms. From PPI interaction networks obtained by integration of information coming from different sources [12, 13] (PPI, literature, expression, evolution, genomic context), functional modules can be identified [14–16] and complexes can be predicted [15, 17–21]. To model the assembly of a complex, it is not relevant to add genetic information as they reflect functional relationships between proteins and not structural interactions.

By taking into account these elements, we developed a strategy that uses protein interaction networks to identify subcomplexes. By querying different databases, we built a non-redundant and un-weighted protein interaction network for each complex. This network is composed of interactions between all single proteins of the complex, named protein subunits, and proteins interacting with any of them. During the biogenesis, protein subunits assemble together to compose subcomplexes that are intermediaries of the entire complex. To identify them, we apply a clustering strategy that groups the subunits having the most interactors in common in the network. The method also allows the identification of proteins potentially involved in the complex biogenesis. It is applicable to any protein complexes even those for which the 3D structure is not yet available. However, the validation of the in silico model requires the 3D structure of the complex.

We first tested our in silico method on two cytoplasmic complexes, the elongator Elp complex [22–24] and the mediator head complex [25–28]. For both complexes, 3D structures are available and the organization of their subunits into subcomplexes is well known. We also tested our method on the *bc1* complex of the respiratory chain because it is located in the inner mitochondrial membrane and moreover one of its subunits is encoded by the mitochondrial genome. The 3D structure of the *bc1* complex has been determined [29] and several models of the assembly process have been proposed [30–32]. The interactions between subunits involved in the early steps of our *in silico* model of the *bc1* complex were experimentally tested.

## Results and discussion

### ISIPS a new in silico approach to identify the subcomplexes of a multiprotein complex

**Fig. 1:**
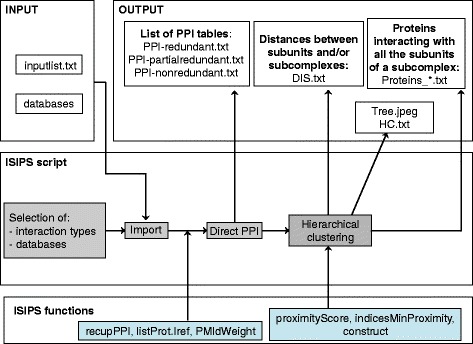
Computational workflow. An overview of the functions and facilities of the script

**Fig. 2:**
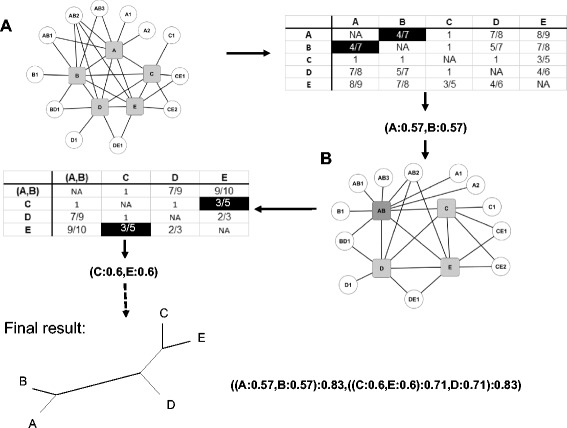
Example of the agglomerative clustering of a PPI network. Panel **a** The initial protein interaction network where nodes represent proteins and links edges, with its associated distance matrix where the smallest distance is highlighted in *black*. It is composed of five subunits: *squares in grey* and 12 proteins directly connected to the subunits: *white circles*. Panel **b** The network after the first round of clustering, its associated distance matrix and the final result

To identify the subcomplexes involved in the biogenesis of a multiprotein complex, we developed ISIPS (In Silico Identification of Protein Subcomplexes), a new approach that identifies subcomplexes, i.e. sets of subunits. The method is based on the assumption that subunits belonging to the same subcomplex interact with the same proteins during the dynamic of assembly. Therefore, in the PPI network, subunits of a subcomplex have more interactors in common than with the other subunits of the complex. These interactors are not only proteins specific of the complex assembly process since some of protein interactions are promiscuous. The workflow of the R script of ISIPS is discribed in Fig. 1. Starting from the list of the subunits which are the components of the studied complex (listed in the input file) and from databases containing all the experimental and predicted PPI of *Saccharomyces cerevisiae* (see Methods), the first step of ISIPS consists of the identification of proteins interacting with the subunits of the complex. The second step of ISIPS consists of an aggregative clustering of the subunits or subcomplexes of the complex based on the PPI network built in the first step. The principle of the clustering is to progressively aggregate the subunits or the subcomplexes having the most interactors in common. The formula of the distance allowing the aggregation of subunits or subcomplexes and the details on the clustering algorithm are given in the Methods section; the result of the clustering is depicted by a tree. For each subcomplex, the list of proteins interacting with all its members is provided. An illustration of the agglomerative clustering approach is given for an artificial network in Fig. 2.

### Characteristics of the network

Our method was applied to three multiprotein complexes of *Saccharomyces cerevisiae* that are composed of subcomplexes or functional modules whose subunit compositions are known**:** the elongator complex (Elp complex), the mediator head complex and the *bc1*complex of the respiratory chain. The list of the subunits of each of these complexes is given in Table 1. From this list, our script ISIPS.R generates the network of the protein interactions of each complex. These PPI are experimental or predicted physical interactions (see Methods) and self interactions of a protein are removed.

**Table 1:** Number of interactions for each subunit of the complexes

Elp sub-unit	Extra PPI	Total PPI	Mediator HEAD sub-unit	Extra PPI	Total PPI	*bc1* sub-unit	Extra PPI	Total PPI
Elp1	2	68	Med6	37	75	Cob	11	26
Elp2	14	52	Med8	28	66	Cor1	9	150
Elp3	3	35	Med11	28	49	Qcr2	12	91
Elp4	1	20	Med17	60	204	Cyt1	13	40
Elp5	1	21	Med18	68	124	Qcr6	28	45
Elp6	0	16	Med20	102	153	Qcr7	30	58
			Med22	112	152	Qcr8	19	38
						Qcr9	26	41
						Qcr10	14	26
						Rip1	28	50

extra PPI are added by using PrePPI database

The network of the Elp complex that is composed of six subunits (Elp1 to Elp6) contained 116 proteins and 197 interactions. The seven subunits of mediator head (Med6, Med8, Med11, Med17, Med18, Med20, Med22) [28] belong to a network composed of 452 proteins and 802 interactions. The network obtained for the *bc1* complex that is composed of ten subunits (Cob, Cor1, Cyt1, Qcr2, Qcr6, Qcr7, Qcr8, Qcr9, Qcr10, Rip1) contains 249 proteins and 522 interactions.

### Identification of the subcomplexes

**Fig. 3:**
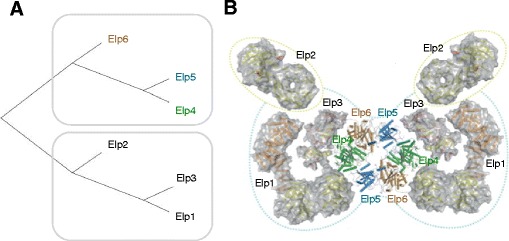
The assembly model of the Elp complex. Panel **a** hierarchical tree representing the distances between the six subunits of the Elp complex. Panel **b** structural model obtained from biological experiments

The results of ISIPS on the identification of the tested subcomplexes presented above are very satisfying. Elp complex is known to be composed of two modules, {Elp1, Elp2, Elp3} and {Elp4, Elp5 and Elp6}; this second module is known to form a dimer that makes a hexameric ATPase [23]. Our script leads to a hierarchical tree with two groups of three subunits that correspond to the two functional modules (Fig. 3). Moreover, in this tree, the subunit Elp2 is separated from the subcomplex Elp1-Elp3, as showed in [23].

**Fig. 4:**
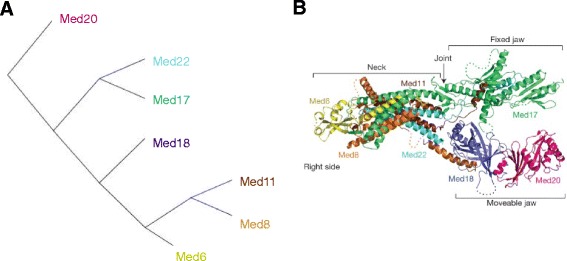
The assembly model of the *Mediator Head* complex. Panel **a** hierarchical tree representing the distances between the seven subunits of the Mediator Head complex. Panel **b** structural model obtained from biological experiments

For the mediator head module, the result of ISIPS is also in broad agreement with the subcomplexes identified biologically [26, 27]. In our model, Med20 is found alone. This result is consistent with the 3D structure of the complex (Fig. 4b) where: Med20 is the most external protein. Med17 and Med22 form a subcomplex, as it has been identified in in-vitro experiments [26], together with Med11, which in our in silico model is found along the subunit Med8. Cross-linking experiments [28] showed that Med11 interacts with Med8, Med22 and Med17. We find a subcomplex composed of Med11 and Med8, probably because these two subunits are buried inside the complex and they have a small number of interactions with proteins not belonging to the complex. Their distance is very low since their partners are mostly the same.

**Fig. 5:**
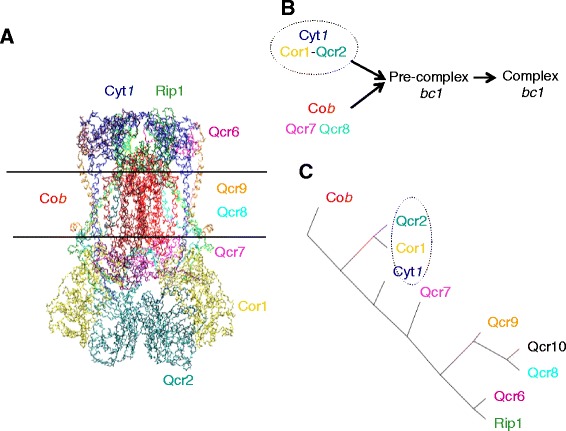
The assembly model of the *bc1* complex. Panel **a** Structure of the *S. cerevisiae bc1* dimer [51] Panel **b** model obtained from biological experiments [29, 30]. The first steps of the assembly process were confirmed by experiments but the order of assembly of subunits Qcr6, Qcr7, Qcr8, Qcr9 and Qcr10 is still undetermined. Panel **c** hierarchical tree representing the distances between the ten subunits of the *bc1* complex. The experimentally validated subcomplex is surrounded by a *grey dotted line*

Results obtained with these two complexes suggest that our software can be used to detect subcomplexes involved in the biogenesis of soluble complexes. The results on the *bc1* complex allows to test whether it can be also used for membrane and/or mitochondrial protein complexes since the *bc1* complex is located in the inner membrane of the mitochondria. More precisely, the 3D structure of the yeast mitochondrial *bc1* complex [29] is composed of nine strongly interconnected subunits that protrude either on the matrix side (Cor1, Qcr2, Qcr7), or into the inter-membrane space side (Cyt1, Rip1, Qcr6) or are imbedded within the inner membrane (Cob, Qcr8, Qcr9) (Fig. 5a), Cob being the first to be co-translationally inserted within the mitochondrial membrane. The tenth subunit, Qcr10 that is not present in the crystal is not essential for the function. A model of the assembly process has been proposed (Fig. 5b) [30–32]. According to this model, the subunit Cob, that is encoded by the mitochondrial genome, together with Qcr7 and Qcr8 would form a first assembly intermediate that would interact with Cyt1, Cor1, Qcr2, Qcr6, Qcr9 to form the pre-complex III, also called the late core (Fig. 5b).

By collecting protein-protein interactions and clustering the subunits of the complex with ISIPS, we obtained a hierarchical tree (Fig. 5c): the subunit Cob is found alone when it is associated with Qcr7 and Qcr8 in the biological models and Cyt1 is found alone when it is associated with Cor1 and Qcr2 in the biological models. In order to understand the discrepancy between the in silico and *in vivo* models, we have tested the *in vivo* model by performing immunoprecipitation experiments in various mutants affecting the assembly of the *bc1* complex.

### Biological experiments to validate the *bc*_*1*_ complex assembly process

**Fig. 6:**
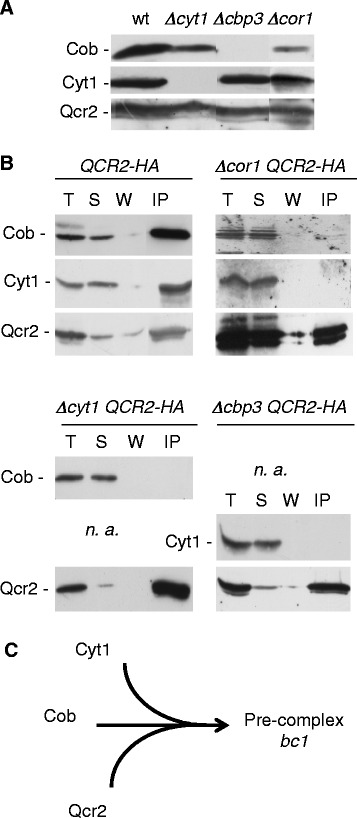
Biological experiments Mitochondrial proteins from wild type (wt) and mutant cells expressing Qcr2-HA were purified. Proteins were resolved on 12% SDS–PAGE followed by immunoblotting with antibodies against Cob, Cyt1 and HA. See list of mutants in Table 2. Panel **a** Accumulation of *bc1* subunits: Cyt1, Qcr2 and Cob, in the wild type and in *Δcyt1*, *Δcor1* and *Δcpb3*. In absence of the translation factor Cbp3, Cob is absent. Panel **b** Mitochondria were solubilised in 1% digitonine and immunoprecipitated with HA coupled agarose beads. The different fractions were analyzed. T: Total mitochondrial proteins; S: supernatant; W: wash; IP: immunoprecipitate. na: not applicable. Qcr2 is subjected to some degradation in absence of Cor1 unless the presence of a cocktail of anti-proteases. Panel **c** Model for the first step of the assembly of complex III

Biological models proposed that Cyt1 might interact with Cor1 and Qcr2 during the first steps of the assembly of the *bc1* complex [31, 32], but the in silico results suggest that Cyt1 is separate from Cor1 and Qcr2. In order to test the interaction between Cob, Cyt1, and Qcr2, we have constructed wild type and mutant strains where Qcr2 is tagged with three copies of the Hemagglutinin (HA) epitope at its C-terminal (Qcr2-HA) and purified mitochondria from these cells. The steady state level of the subunits Cob, Cyt1 and Qcr2 was analyzed by Sodium Dodecyl Sulfate PolyAcrylamide Gel Electrophoresis (SDS-PAGE) and western blot in the wild type and three mutants, each abolishing the synthesis of either Cyt1 (*Δcyt1),* or Cor1 *(Δcor1)* or Cob (*Δcbp3*) Fig. 6a. Cbp3 is essential for the translation of the *COB* mRNA. Thus Cob is not detected in the *Δcbp3* mutant. In *Δcyt1* and *Δcbp3*, the steady state level of Qcr2 is as in the wild type (Fig. 6a). In *Δcor1*, some proteolysis of Qcr2-HA occurs suggesting that Cor1 protects Qcr2 from proteolysis. The steady state level of Cyt1 is stable in absence of Cob or Cor1, and the amount of Cob decreases in the absence of Cyt1, or Cor1, suggesting that these subunits protect Cob from the degradation.

In order to determine if there are some physical interactions between the subunits in the different mutant contexts, we performed immunoprecipitation experiments using the HA tag (Fig. 6b). As expected, in the wild type control the four subunits (Cob, Cyt1, Qcr2) are pulled down together. However, in the absence of Cyt1 (*Δcyt1*), although a substantial level of Cob is still detected in SDS-PAGE analysis, it no longer co-immunoprecipitates with Qcr2 suggesting that Cob does interact stably with Qcr2 in absence of Cyt1. Reciprocally, in the absence of the Cob (*Δcbp3*), although the steady state level of Cyt1 and Qcr2 is not decreased as compared to wild type, Cyt1 no longer co-immunoprecipitates with Qcr2 suggesting that Cyt1 does not stably interact with Qcr2 in absence of Cob. Finally, in the absence of Cor1, neither Cob, nor Cyt1 interact with Qcr2. Thus the three subunits appear to be interdependent for their assembly and no subcomplex Cyt1-Qcr2 is formed in absence of Cob. Thus, the immunoprecipitation experiments are consistent with the prediction of the in silico model regarding the first steps of the assembly of the *bc1* complex (compare Figs. 5a and 6c).

It would be also interesting to test *in vivo* the existence of the subcomplex Cob/Qcr7/Qcr8 proposed by the biological model and not found by the in silico model. We plan to carry out these experiments in the future when antibodies against the two small subunits Qcr7 and Qcr8 will be available.

### Comparison of the results obtained using different similarity scores

Different similarity scores (http://elib.uni-stuttgart.de/bitstream/11682/2573/1/Evert2005phd.pdf) have been tested to find subcomplexes and their clustering results have been compared. For the three complexes (Elp, mediator head and *bc1*) the pseudo-Jaccard index gives the in silico model that is the nearest to the biological models and our biological results.

For the Elp complex, the Dice score (http://elib.uni-stuttgart.de/bitstream/11682/2573/1/Evert2005phd.pdf) gives the same clustering results as the pseudo-Jaccard index because the number of proteins that interact with two subunits is low. The other similarity scores do not distinguish the two functional modules (Elp1, Elp2, Elp3 vs Elp4, Elp5, Elp6).

For the mediator head, the Dice score and the pseudo-Jaccard index are the only similarity scores that give a model where Med20 is the last subunit to be added, which fits well with the 3D structure of the mediator head. The difference between these two models (Additional file 1: Figure S1) is that the location of the subunit Med11 in the tree, is separated from the subunit Med8 using the Dice score, while both subunits are in the same subcomplex using the pseudo-Jaccard index. Still, it is difficult to decide between these two models in view of the 3D structure of the mediator head.

For the *bc1* complex, the three models obtained by using the Dice, Minimum Sensitivity (MS) and pseudo-Jaccard scores are very similar except for the subunit Qcr7 that is split from Qcr9 in the tree obtained using the Dice score (Additional file 1: Figure S1). None of these three models match the biological models [30–32] where Qcr7 and Qcr8 form an early core subcomplex. Indeed, Qcr8 shares more protein partners with Qcr10 than with Qcr7; moreover Qcr7 interacts with many more proteins than Qcr10 and the chance that it is in the same subcomplex as Qcr8 is lower than the chance of Qcr10 and Qcr8 being in the same subcomplex. We can also note that Qcr7 interacts with a lot of proteins of the OXidative PHOSphorylation (OXPHOS) complexes, that is not the case of Qcr10.

### Effect of the false and true positives on the in silico model

Proteomic experiments often provide incomplete data due to the difficulty of detecting some PPI. Since it is impossible to know if a PPI is missing or if it does not really exist, the two cases are indistinguishable in the network and considered as a negative. In the network the PPI correspond to stable interactions because the transient interactions are more difficult to identify experimentally. Thus, as these interactions are often missing in the network, our algorithm can be biased towards the most stable subcomplexes. To limit the effect of these missing PPI, interactions predicted by combining structural and non-structural information are added (see Methods). However, these predicted interactions may contain false PPI that could be then filtered when their confidence score is lower than a relevant cut-off (see the section “Choice of the cut-off of confidence score”). False PPI can also be introduced by different kinds of experiments [7]. They cannot be filtered out because all databases do not provide confidence scores and when those are available, they are not computed in the same manner [12, 33, 34]. Moreover, it is important to note that two interacting proteins detected by “physical” methods are just identified in a same molecular assembly and are not systematically in contact. Then, many interactions are due to the steric overcrowding and to some proteins that are more abundant and/or more stable and/or more stiky than others. The pseudo-Jaccard index used as proximity score to cluster the subunits is well adapted to take these kind of drawbacks into account (see section methods).

### Robustness of the algorithm to the update of the databases

It is important to note that due to the high number of false positive and false negative PPI in the databases, each update can change the protein interaction network of the complex of interest. Some interactions can be added and other removed, which may change the probability of two subunits (or subcomplexes) to form a subcomplex and then alter the results of the clustering process.

The in silico models of the assembly of three complexes (Elp Mediator head and *bc1*) were not affected by the update of the databases during the 18 months of this study, despite changes in the networks. That would not be the case if the network was clustered with graph partition methods based on the density (MCODE [35] and ClusterOne [36]) or on the modularity (Tfit [37]).

### Choice of the cut-off of confidence score of the interactions from PrePPI database and impact on the subcomplexes identified with ISIPS

To complement the PPI network, predicted PPI from the PrePPI database [38, 39] are added to the experimental PPI. We choose this database because it integrates a high number of criteria combined to provide confidence scores. When the biogenesis of the complex is partially known, a cut-off on the predicted PPI score can be adjusted to remove the less reliable PPIs and at the same time to keep PPIs known to modify the biogenesis of the studied complex. The default cut-off of the database PrePPI is equal to 0.5. For the Elp complex, the tree representing the distance between the subunits of the complex is always the same irrespective to the cut-off on the PrePPI score because the number of the added interactions remains low (Table 1). But for the mediator head complex, the distance between the seven subunits of the complex changes depending on the cut-off value. We have chosen a cut-off value equal to zero, i.e. no filtering of the predicted PPI. Indeed, the mediator head complex being linked with the RNA polymerase II and transcription factors [40], interactions involving the subunits of these two complexes have then to be added in the network though they have low scores due to the lack of evidence sources used for the prediction. For the *bc1* complex, when we lower the cut-off value, we get proteins involved in general processes such as members of the chaperone family. While with the cut-off value equal to 0.5, most of the added interactions are located in mitochondria and were not described previously.

When the predicted PPI are not added to the experimental PPI, all the subcomplexes detected by the experiments are not in agreement with those found in silico. This is also the case if one uses only the predicted PPI.

### Weakness of our method

We saw previously that some subcomplexes of the *bc1* complex identified with ISIPS, contradict the biological models described in the literature: The subunit Cob is found alone when it is associated with Qcr7 and Qcr8 in the biological models [30–32]. This result may be explained by the fact that mitochondrially-encoded Cob is the most hydrophobic subunit of the *bc1* complex (8 transmembrane segments) and might be more difficult to identify in mass spectrometry experiments. Indeed, we remarked that in the databases it does not interact with the same proteins as the other subunits.

Another issue with the *bc1* complex is the fact that some subunits of the complex are more stable than others. The subunits inside the membrane are less stable because if they are not quickly assembled, they are degraded because they can disrupt the cohesion of the membrane. However, our algorithm is based on stable interactions since the instable interactions are not present in the network. Then, it does not allow us to identify subcomplexes formed by subunits located inside the membrane, which are less stable than the other subunits.

The PPI between subunits of a complex could be weighted by the measure of their stability, but the different published results [41, 42] are not mutually consistent because the experimental conditions used are different. Hence, they cannot be used in the distance computation.

## Conclusion

Many tools have been developed to identify protein complexes and protein functional modules from static or dynamic PPI networks [43, 44]. To refine the detection of the complexes, network clustering algorithms are still being developed [45] but so far, there is no method of this type to study their biogenesis. We developed ISIPS, a tool to identify subcomplexes and proteins involved in the building of the *Sacchoromyces cerevisiae* and other species complexes. To validate our approach, we choose three complexes for which the structure and subcomplexes are already known: Elp, mediator head and *bc1*. They belong to the small number of complexes for which subcomplexes have been described by different kinds of experiments.

ISIPS proposes different proximity scores to cluster the subunits in subcomplexes, but we show for the three examples that the pseudo-Jaccard index gives the clustering results the closest to the biological models.

ISIPS is an approach that is based only on a PPI network. It does not take abundance, half-life, affinity and localization of proteins into account. However, these data are important since PPI depend on the number of occurrences of proteins at a precise time and place. To understand the in silico results, such information must be taken into account a posteriori.

Another aspect to keep in mind is that some complexes interact strongly with other complexes to form supercomplexes. Thus, when a complex is part of a supercomplex, the network built from its proteins involves interactions with the supercomplex proteins that influence the in silico model of the complex biogenesis. Therefore, it does not always reflect what is happening biologically.

The new approach presented in this article, despite its limitations, can help biologists to choose experiences that allow them to describe the assembly process of a complex. It also permits to reduce the combinatorial space for modeling and simulation interfaces between all the subunits of a complex.

## Methods

Our aim is to extract information from the protein interactions network involving subunits of a protein complex in order to identify the subcomplexes that form it. The different steps of the methodology are: 1) the construction of the PPI network; 2) the identification of subcomplexes; 3) the identification of proteins interacting with the subcomplexes.

### Construction of the protein-protein interaction network

To build the network, we used the interactions involving the subunits of the complex of interest from the 14^*th*^ release of iRefIndex [46] that collects data from eight primary databases. We split the file (in our case: 559,292.mitab.04072015) into eight “database” files stored in a unique folder. Within each database file, Taxon A and B must be the same, else the corresponding interactions are removed. We also used the most recent version of some frequently updated databases such as BioGRID [47] and Intact [33]. In addition, we used the PrePPI database [39] a repository that provides predicted or experimental PPI with associated confidence scores. Indeed, we needed to add predicted PPIs because some subunits of protein complexes do not have any partner in common with the other subunits and therefore would not be included in the model. The PPI involving the proteins of the studied complex are downloaded from the site: https://honiglab.c2b2.columbia.edu/PrePPI/ and those having a confidence score greater than a cut-off (0.5 by default) are stored in a file in the same format as the other database files (see Additional file 2: Table S1).

To construct and cluster the PPI network of the complex of interest, we developped the R script ISIPS.R which workflow is described in Fig. 1. This script inputs a list of Uniprot identifiers (ID) of the proteins subunits of the complex of interest, stored in the “inputlist.txt” file. The script also requires specifying the directory containing the previously mentioned databases. First, we search for interactions involving the proteins of the studied complex (predicted or experimentally identified by physical methods) and we discard self interactions. Three text files are generated: one with the complete list of interactions, one with partially redundant interactions (PPI with the same publication ID are removed) and one non-redundant. The network is unweighted but the number of Pubmed IDs corresponding to each column is indicated in the last file. This information is a criterion of reliability of the interaction that is not used here.

Source code of ISIPS.R, examples and guides for the complexes presented in this article are available on: http://bim.igmors.u-psud.fr/isips.

### Identification of the subcomplexes involved in the assembly of the complex

Our method to identify subcomplexes involved in the assembly of the complex is based on the hypothesis that within the network, assembly intermediaries are represented by subgraphs of strongly interconnected proteins. Indeed, the proteins of a subcomplex share more partners between them than with the rest of the network. Two subunits and/or subcomplexes are “close” as they share more partners, by “close” we mean that they have a high probability to form a subcomplex.

Then, to evaluate the probability of two subunits (or subcomplexes) *x* and *y* to form a subcomplex, we compute *j*(*x*,*y*), a pseudo-Jaccard similarity index between *x* and *y, defined as:*

$$ j\left( x, y\right)=\left( N\left( X\cap Y\right)\right)- N\left({SU}_x\cap {SU}_y\right)/ N\left( X\cup Y\right)- N\left({SU}_x\cup {SU}_y\right) $$

where *X* is the set of proteins interacting with the subunit (or the subcomplex) *x*; *Y* is the set of proteins interacting with the subunit (or the subcomplex) *y; N(X∩Y)* is the number of proteins that interact with *x* and *y; N(X⋃Y)* is the number of proteins that interact with *x* or *y*; *SU*_*x*_ and *SU*_*y*_ are the number of subunits of the complex interacting with *x* and *y* respectively; *N(SU*_*x*_ *∩SU*_*y*_ *)* is the number of subunits interacting with *x* and *y*; *N(SU*_*x*_ *⋃ SU*_*y*_ *)* is the number of subunits interacting with *x* or *y.*

Note that this metric is not exactly the Jaccard index of the neighbours of proteins belonging to the complex, because we choose to ignore the interactions within the complex by subtracting in our formula the number of subunits of the complex interacting with *x* and/or *y*. It is actually the relative number of interactants external to the complex and sharing two subunits (or subcomplexes) that allows to compute their chance to belong to a same subcomplex.

For the identification of the subcomplexes involved in the assembly of the complex, an agglomerative clustering is used, which requires a distance measure (see Fig. 2). This distance *d(x,y)* is equal to *1-j(x,y).* As an example, in Fig. 2, the distance *d(x,y)* between subunits *A* and B is equal to *d*(*A,B*) = 1-(6-3)/(10-3) = 4/7.

### Advantage of the distances between sub-units and/or sub-complexes used in ISIPS

The pseudo-Jaccard index presented above is a similarity score between two sets easy to compute and to understand. In our case, we can simply describe the pseudo-Jaccard index between two subcomplexes (or subunits) as the proportion, among all proteins outside the complex interacting with at least one of the two subcomplexes (or subunits), of proteins that interact with both. One of the advantages of this index is to be inherently symmetric. Furthermore, it will be more favorable to pairs of subcomplexes (or subunits) that have a small number of interacting proteins. On the contrary, if one of the two subcomplexes (or subunits) has a lot of interacting proteins, a big proportion of these proteins, which are not involved in the complex assembly, can be considered as noise since they cannot guide our task of complex assembly. Therefore, using a formula that penalizes these kinds of subcomplexes (or subunits) with a lot of interacting proteins, it allows to focus on those whose neighborhood in the PPI graph carries the most relevant information.

In order to allow users to compare this index with other alternatives, we have included five other statistical formulas to compute similarity between two subcomplexes in ISIPS. These formulas depend on the sizes of 4 sets of proteins outside the complex (proteins interacting with none of those two subcomplexes, with only the first one, with only the second one, or with both of them). They can be normalized between zero and one. Introduced in various contexts and summarized in a unified formalism (http://elib.uni-stuttgart.de/bitstream/11682/2573/1/Evert2005phd.pdf), they are described in details in the user manual of ISIPS (http://bim.igmors.u-psud.fr/isips).

The clustering algorithm developed to identify the sequence of subcomplex by using the similarity scores defined above is a bottom-up agglomerative clustering. Our motivation to use this algorithm is the assumption that the proteins or subcomplexes with the most specific common interacting proteins have a higher probability to be in a same subcomplex. On the contrary, proteins interacting with a lot of proteins unrelated with the complex will have more interacting possibilities in the cell, and will therefore presumably not be involved in the first subcomplexes. First, the algorithm starts by computing the distances between all the subunits and then groups the subunits having the smallest distance. This cluster is considered as the first subcomplex in the model. Second, it computes the distances between this first subcomplex and the remaining subunits. Third, it replaces in the previous distance matrix the lines and the columns corresponding to the subunits of the subcomplex by the distances between the subcomplex and the other subunits. Previous steps are iteratively repeated to gradually aggregate the subunits. This process terminates when all subunits are (see Fig. 2b).

### Results obtained with the ISIPS method

The script generates different types of results:

1. The distance matrices computed at each step of the clustering are stored successively in a file.
2. The result of the clustering, stored in a file having a linear format where two aggregated subunits or group of subunits are written between parentheses and separated by a comma. The distance between the two aggregated elements is indicated after the elements and separated by a colon. The groups of subunits (or subunit alone) are ordered according to the progress of their formation, from the left to the right.
3. A graphical representation of proximities between the subunits and/or subcomplexes. We choose to represent the results of our clustering method by a rooted dendrogram (the root is the final complex), drawn horizontally in order to have enough space to display the labels representing the subunits. We warn the users that the vertical ordering of the subunits, or the clusters, should not be interpreted as a chronological ordering. Therefore, we also provide the user with the corresponding tree in a text file, so that it can be used in any representation tool with a different vertical ordering, in order to represent a hypothesis on the order of subcomplex assembly. Branches separating two sets A and B of subunits are long if they are strongly supported by the proximity matrix, that is if two elements within the same subunit (A or B) are expected to have a larger proximity index than two elements in different subunits. More precisely, the length of a branch which separates A and B is set to the rate of well designed triples [48] that is the proportion of sets {*x,y,y’*}, with *x* in A and *y* and *y*’ in B (or *x* and B and *y* and *y*’ in A) such that *j*(*y*, *y* ^′^) ≥ max(*j*(*x*, *y*), *j*(*x*, *y* ^′^)), where *j* is the proximity index between subunits presented above.
4. A list of proteins interacting with at least two proteins of the subcomplex.

### Strains construction

Each deletion mutant was constructed in the CW252 strain that has the W303 nuclear background *MAT alpha ade2-1 ura3-1 his3-11,15 trp1-1 leu2-3112 can1-100* (see Table 2).

**Table 2:** List of the strains used

Name	Genotype	Origin
Cor2-HA	*Cor2-HA-Sphis5*	This work
Cor2-HA*Δcyt1*	*Cor2-HA-Sphis5 cyt1::LEU2*	This work
Cor2-HA *Δcbp3*	*Cor2-HA-Sphis5 cbp3::G418*	This work
Cor2-HA *Δcor1*	*Cor2-HA-Sphis5 cor1::HIS3*	This work
Δbcs1	*bcs1::URA3*	[52]

Qcr2 was tagged at its C-terminus with the HA epitope using the *Schizosaccharomyces pombe* marker gene *his5* (*Sphis5* complements the *S. cerevisiae* mutation *his3*) as described in [49].

In each case, the correct integration of the mutation or the tag was confirmed by PCR amplification and sequencing. We verified that the tags did not modify the respiratory phenotype.

### Biochemical analyses

Mitochondrial proteins from wild type and mutant cells expressing Qcr2-HA were isolated from galactose grown cells by differential centrifugations after digestion of cell walls with Zymoliase-100 T [50]. For immunoprecipitation experiments, mitochondrial proteins were solubilised in 1% digitonine. The suspension was centrifuged for 15 min at 4 °C at 100000 g. Monoclonal HA antibodies coupled agarose beads (Sigma) were added to the supernatant. Samples were incubated under gentle shaking for 90 min at 4 °C in presence of PhenylMethaneSulfonylFluoride (PMSF) and the complete protease inhibitor cocktail (Roche). The beads were washed twice with the lysis buffer and the different fractions were analyzed by SDS-PAGE and western blotting experiments. Proteins were separated on 12% SDS–PAGE followed by immunoblotting with antibodies against Cob, Cyt1, HA. Polyclonal antibodies against Cob and Cyt1 were raised in the laboratory and used at 1/10,000. The monoclonal anti body anti-HA (1/10,000) was from Santa Cruz Biotechnology.

## Additional files

Additional file 1: Figure S1. Assembly models obtained with different similarity scores. Panel A: hierarchical tree representing the distances between the seven subunits of the *Mediator Head* complex. Upper Part: tree obtained with the Dice similarity score. Lower part: tree obtained with the pseudo-Jaccard similarity score. Panel B: hierarchical trees representing the distances between the ten subunits of the *bc1* complex. Upper part: tree obtained with the Dice similarity score. Lower part: tree obtained with the MS or pseudo-Jaccard similarity score. (PDF 107 kb)

Additional file 2: List of the proteins interacting with the subcomplexes of the studied complexes. The file includes three sheets, one for each complex: ELP, mediator head and *bc1*. (XLS 32 kb)

## Abbreviations

HA: Hemagglutinin
ID: Identifier
ISIPS: In Silico Identification of Protein Subcomplexes
MS: Minimum sensitivity
OXPHOS: OXidative PHOSphorylation
PMSF: PhenylMethaneSulfonylFluoride
PPI: Protein-protein interaction
SDS-PAGE: Sodium Dodecyl Sulfate PolyAcrylamide Gel Electrophoresis
