# Supplementary figures and images for: Development of an in silico method for the identification of subcomplexes involved in the biogenesis of multiprotein complexes in Saccharomyces cerevisiae
Source: BMC Syst Biol. 2017 Jul 11;11:67. doi: 10.1186/s12918-017-0442-0 (PMC5504824; doi:10.1186/s12918-017-0442-0)

**A**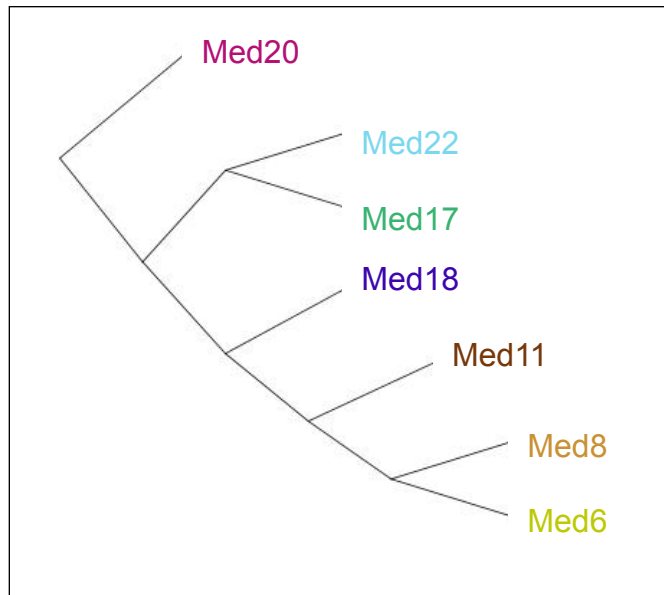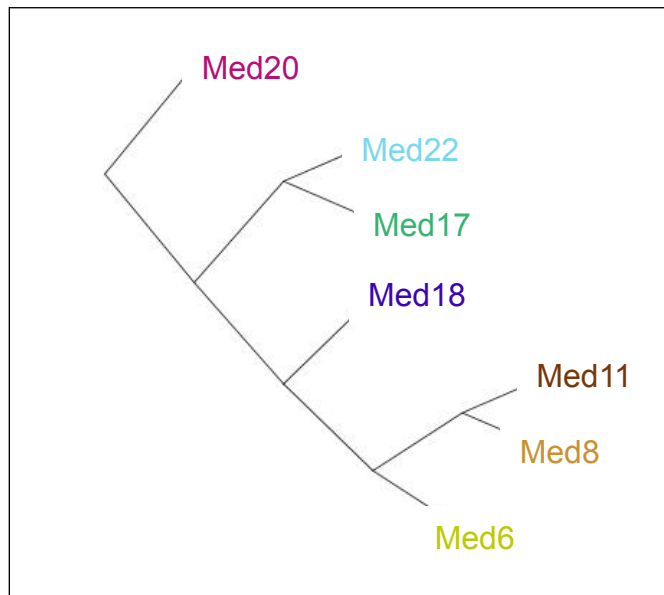**B**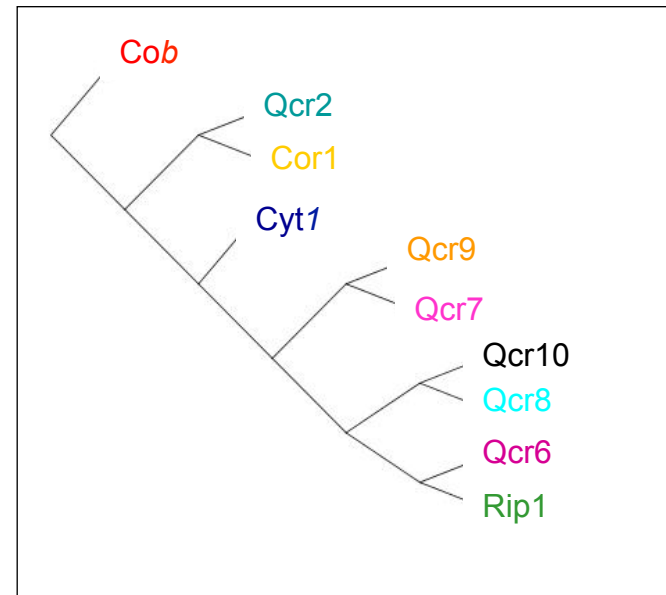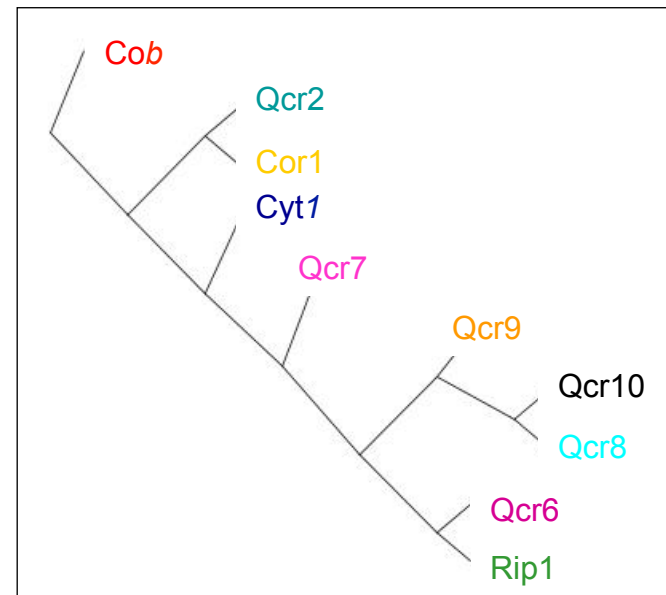

Supplement: Supplementary file 1 — Assembly models obtained with different similarity scores. Panel A: hierarchical tree representing the distances between the seven subunits of the Mediator Head complex. Upper Part: tree obtained with the Dice similarity score. Lower part: tree obtained with the pseudo-Jaccard similarity score. Panel B: hierarchical trees representing the distances between the ten subunits of the bc1 complex. Upper part: tree obtained with the Dice similarity score. Lower part: tree obtained with the MS or pseudo-Jaccard similarity score. (PDF 107 kb) [file 12918_2017_442_MOESM1_ESM.pdf]
